# Genomic and Physiological Investigation of Heavy Metal Resistance from Plant Endophytic *Methylobacterium radiotolerans* MAMP 4754, Isolated from *Combretum erythrophyllum*

**DOI:** 10.3390/ijerph18030997

**Published:** 2021-01-23

**Authors:** Mampolelo M. Photolo, Lungile Sitole, Vuyo Mavumengwana, Matsobane G. Tlou

**Affiliations:** 1 Department of Biochemistry, Faculty of Science, Auckland Park Campus, University of Johannesburg, Johannesburg 2092, South Africa; empeena@gmail.com (M.M.P.); lsitole@uj.ac.za (L.S.); 2 DST-NRF Centre of Excellence for Biomedical Tuberculosis Research, South African Medical Research Council Centre for Tuberculosis Research, Division of Molecular Biology and Human Genetics, Faculty of Medicine and Health Sciences, Tygerberg Campus, Stellenbosch University, Cape Town 7505, South Africa; vuyom@sun.ac.za; 3 Department of Biochemistry, School of Physical and Chemical Sciences, Faculty of Natural and Agricultural Sciences, Mafikeng Campus, North-West University, Mafikeng 2790, South Africa

**Keywords:** *Methylobacterium radiotolerans*, endophyte, comparative genome analysis, maximum tolerance concentration, transmission electron microscopy

## Abstract

*Combretum erythrophyllum* is an indigenous southern African tree species, a metal hyperaccumulator that has been used as a phytoextraction option for tailing dams in Johannesburg, South Africa. In hyperaccumulators, metal detoxification has also been linked or attributed to the activities of endophytes, and, in this regard, metal detoxification can be considered a form of endophytic behavior. Therefore, we report herein on the identification of proteins that confer heavy metal resistance, the in vitro characterization of heavy metal resistance, and the production of plant growth-promoting (PGP) volatiles by *Methylobacterium radiotolerans* MAMP 4754. Multigenome comparative analyses of *M. radiotolerans* MAMP 4754 against eight other endophytic strains led to the identification of zinc, copper, and nickel resistance proteins in the genome of this endophyte. The maximum tolerance concentration (MTC) of this strain towards these metals was also investigated. The metal-exposed cells were analyzed by transmission electron microscopy (TEM). The ethyl acetate and chloroform extracts (1:1 *v*/*v*) of heavy metal untreated *M. radiotolerans* MAMP 4754 were also screened for the production of PGP compounds by Gas Chromatography–Mass Spectroscopy (GC/MS). The MTC was recorded at 15 mM, 4 mM, and 12 mM for zinc, copper, and nickel, respectively. The TEM analysis showed the accumulation of metals in the intracellular environment of *M. radiotolerans* MAMP 4754, while the GC/MS analysis revealed several plant growth-promoting compounds, including alcohols, phthalate esters, alkenes, ketones, sulfide derivatives, phenols, and thiazoles. Our findings suggest that the genetic makeup of *M. radiotolerans* MAMP 4754 encodes heavy metal resistant proteins that indicate hyperaccumulator-specific endophytic behavior and the potential for application in bioremediation. The production of plant growth-promoting volatiles in pure culture by *M. raditotolerans* MAMP 4754 is a characteristic feature for plant growth-promoting bacteria.

## 1. Introduction

Heavy metals are nondegradable, inorganic contaminants that are extremely toxic to many different living organisms [1]. Copper, zinc, iron, manganese, nickel, and cobalt are essential elements, which can be toxic in large amounts, whereas cadmium, mercury, lead, and silver are nonessential and extremely toxic elements [2,3,4,5]. The most common heavy metals, which are contributors to soil toxicity, are cadmium, nickel, lead, copper, manganese, and zinc [1,6]. The major environmental causes of heavy metal emissions include mining, agricultural pesticides, pharmaceuticals, sewage sludge, and industrial activities [7,8,9]. Conventional methods such as chemical precipitation, ion exchange, or reverse osmosis for the elimination of heavy metals from contaminated soils are costly and pose a variety of challenges, including high reagent requirements and the production of toxic sludge [10,11,12]. On the contrary, environmental remediation processes, such as phytoremediation, provide low-cost benefits, lower reagent requirements, and decreased production of disposable sludge [13,14].

Phytoremediation is the utilization of plants in the accumulation, degradation, or removal of contaminants, such as heavy metals, from the environment [15,16]. Plants with these remarkable metal remediating abilities are known as hyperaccumulator plants [17], and examples of such plants include *Combretum erythrophyllum*, a native southern African tree found in rivers and wetlands. This plant species has been used in the management of seepage from AngloGold Ashanti Ltd. (Johannesburg, South Africa) gold mine tailings [18] and, therefore, is generally considered a suitable candidate for the control of seepage and in situ stabilization of heavy metals [19]. Phytoremediation of soil contaminated with heavy metals can be achieved by various mechanisms, which include phytoextraction, phytovolatization, phytostabilzation, rhizodegradation, phytohydraulics, and phytodegradation [8,13,20,21,22,23,24]. Plant growth-promoting endophytes have been documented to play a significant role in the phytoremediation of heavy-metal-contaminated soil [25,26,27,28,29]. The partnership between endophytes and host plants enables adjustments in the physiology and metabolism of the plant [30,31]. Plant growth-promoting endophytes are able to detoxify pollutants and relieve planta stress through metabolic pathways, which are encoded by genes involved in nitrogen fixation, phytohormone synthesis, and degradation of contaminants. Examples of the enzymes/proteins involved include cytochrome P450 monooxygenases, hydrolases, polyphenol peroxidases, heavy metal ATPases, metallothioneins, and transporter proteins [32,33,34,35,36]. Furthermore, increasing evidence suggests that endophytes produce phytohormones, which also play an important role in accelerating heavy metal detoxification in plants [37,38]; as well as producing phosphatases, which are known as plant growth promotion traits [39].

The strain of interest in this study, *Methylobacterium radiotolerans* 4754, is an endophytic isolate from *C. erythrophyllum* that was isolated and identified as previously reported in [40]. Therefore, we hypothesize that this strain possesses heavy metal tolerance properties that are complementary to life inside the hyperaccumulator host plant and also produces secondary metabolites/volatiles that play a role in plant growth promotion, a characteristic feature for beneficial endophytic behavior.

Plant growth-promoting traits for endophytes can be identified by the presence (in the genomes) of genes that are implicated in the enhancement of nutrient absorption/availability, amelioration of oxidative and abiotic stresses, and catabolism of aromatic compounds [41]. Furthermore, for hyperaccumulator-inhabiting endophytes, the presence of genes/proteins for heavy metal resistance can be considered to indicate a specialized role played by the endophyte within the host plant. The latter is supported by the reported isolation from hyperaccumulator plants of metal-resistant bacterial isolates capable of detoxification and translocation of several heavy metals, including cadmium, nickel, copper, zinc, and mercury [42].

In this study, the reciprocal smallest distance (RSD) algorithm-based comparative multigenome analysis of *M. radiotolerans* MAMP 4754 versus selected Proteobacteria species (endophytic and rhizospheric) was employed for the investigation of genes/proteins possibly implicated in plant-beneficial behavior. We also report on the heavy metal detoxification properties and the production by this strain of organic compounds that are involved in plant growth promotion.

## 2. Method and Materials

### 2.1. Comparative Genome Analysis

Comparative genome analysis was done as per the methods described in [43]. Genome coding sequences were compared using the RSD algorithm with the most stringent blast E-value (1 × 10^−20^) and divergence thresholds (0.2) [44]. RSD makes use of reciprocal best blast hits (RBHs), global sequence alignment, and the highest probability estimation of evolutionary distance in the identification of orthologs between two genomes or two input files [44]. The complete genomes of endophytic and nonendophytic plant growth-promoting Proteobacteria were used (Table 1), and gene products putatively responsible for endophytic behavior were predicted. First, a nonendophytic (*Methylobacterium nodulans* ORS 2060) and an endophytic bacterium of the same genus (*Methylobacterium mesophilicum* SR 1.6/6) were compared. Orthologs were identified in both genomes, and the genes unique to the nonendophyte were subtracted from the genome of the endophytic bacterium *M. mesophilicum* SR 1.6/6. These orthologs were also subtracted from *M. radiotolerans* MAMP 4754, leaving behind genes putatively responsible for endophytic behavior. Furthermore, the common putative endophytic genes were compared to the genomes of eight different endophytic bacteria (Table 1), using the RSD algorithm in order to ensure their presence in several endophytic bacteria and their confirmation as candidates for endophytic behavior.

### 2.2. Growth Profile of M. Radiotolerans MAMP 4754

The bacterium, *M. radiotolerans* MAMP 4754, was initially cultured on Luria broth (LB) agar, incubated at 28 °C overnight, and a single colony was selected and inoculated into 10 mL of LB broth (preinoculum). The exponential phase preinoculum (10 mL) was used to inoculate LB medium (1000 mL) in a shake flask, and the growth was monitored at regular intervals by measuring the optical density at 600 nm in a spectrophotometer (Shimadzu spectrophotometer UV-1800; Shimadzu Corporation. Analytical & Measuring Instruments Division, Kyoto, Japan).

### 2.3. Determination of Maximum Tolerance Concentration (MTC)

The maximum tolerance concentration (MTC) for *M. radiotolerans* MAMP 4754 was determined for the heavy metals zinc, nickel, and copper (ZnCl_2_.6H_2_O, NiCl_2_.6H_2_O, and CuSO_4_.5H_2_O). The heavy metal stocks were prepared in deionized water, sterilized by a filter membrane with a pore size of 22 µm, and stored at 4 °C until used. Each heavy metal solution was added to Luria broth (LB) at varying final concentrations ranging from 1 mM to 15 mM for zinc, nickel, and copper. Bacterial growth was determined spectrophotometrically at OD_600nm_ [57].

### 2.4. Transmission Electron Microscopy

In order to investigate the fate of the heavy metals in the cell, transmission electron microscopy (TEM) was performed on the metal-treated and untreated cells. Briefly, the pellets were washed three times with phosphate-buffered saline (PBS) followed by their resuspension in 1 mL of fixative solution (2.5% glutaraldehyde and 2% formaldehyde), and kept at 4 °C for 6 h. Post fixation, the samples were centrifuged, and the pellets were washed with PBS five times. Finally, the pellets were resuspended in 1 mL of sodium phosphate buffer and analyzed by TEM. Studies were performed at an acceleration voltage of 200 kV using a Jeol JEM-2100F Field Emission Microscope instrument equipped with an LaB6 source. The TEM samples were prepared by dropping 10 µL of the resuspended pellets on a 200-mesh size copper grid coated with a lacy carbon film. The images were verified using a digital charge-coupled device camera.

### 2.5. Gas Chromatography HighResolution Time-of-Flight Mass Spectrometry Analysis

In order to investigate the production of plant growth-promoting volatiles from *M. radiotolerans* MAMP 4754, an extraction was performed from the cell culture as previously described by [40]. The extract was studied for metabolites/volatiles using gas chromatography high-resolution time-of-flight mass spectrometry (GC-HRTOFMS (LECO Corporation St. Joseph, MI, United States)), operating in high resolution, equipped with a Gerstel MPS multipurpose autosampler (Gerstel Inc., Mülheim, Germany). For the analysis, the samples were run in a 30 m × 0.25 mm capillary column with a film thickness of 0.25 µm. The carrier gas was helium, and it was maintained at a column flow rate of 1 mL/min. A 1 µL sample of the extract was injected and the column temperature was maintained at 75 °C, followed by temperature programming at 10 °C/min to 235 °C for 2 min, and finally to 300 °C at a rate of 40 °C/min for 3 min (scan range: 45–500 m/z). The mass spectrometer and transfer line were held at 250 °C. Peak picking, peak and retention time alignment, as well as detection and matching were done on ChromaTOF-HRT^®^ software (LECO Corporation, St. Joseph, MI, United States). A signal-to-noise (S/N) ratio of 100 was used and the similarity/probability match was >70%, before a name was assigned to a compound using Mainlib, NIST, and the Feihn metabolomics database through comparison of the mass spectra data, the molecular formula, as well as the retention time.

## 3. Results and Discussion

### 3.1. Comparative Multigenome Analysis, MTC, and TEM Analysis

The use of comparative multigenome analysis is valuable in understanding the genetic and metabolic diversity of related microbes involved in different types of interactions with plants as well as with animals [58]. It also gives insight into various genomes from different bacteria encoding similar functions. Thus, the comparison of genes encoded by the endophytic *Methylobacterium* strain to the genomes of a rhizospheric strain of the same genus aids in the identification of genes specifically involved in endophytic behavior within the endophytic strain. Upon RSD-based analysis with eight plant growth-promoting endophytes of phylum Proteobacteria genes/gene products that play a role in the transport, secretion, and delivery system, we identified plant polymer degradation, transcriptional regulation, redox potential maintenance, and detoxification functions.

Interestingly, from the comparative analysis, proteins that confer resistance to zinc, copper, and nickel (heavy metals) (Table 2) stood out in the genome of *M. radiotolerans* MAMP 4754, possibly indicating involvement in endophytic behavior and/or a specialized role for the bacteria in the hyperaccumulator host plant. A probable explanation for the presence of these proteins is that the bacterium protects the plant from heavy-metal-induced stress and/or augment the process of metal detoxification by the host plant. Heavy metal stress can induce the production of ethylene and an excess of ethylene can inhibit plant development [59]. In this study, copper resistance gene A (Table 2), reportedly the main genetic element for copper resistance in Gram-negative bacteria [60,61], was also identified from the genome of *M. radiotolerans* MAMP 4754. Furthermore, TonB-dependent, transporter-related genes were also identified in the analysis (Table 2). These are outer membrane proteins that are frequently found binding and transporting nickel and iron chelates and, thus, play a vital role in bacterial metal transport [62,63]. Additionally, the nickel-responsive transcriptional regulator protein (NikR) (also identified in the comparative analysis) has been reported to regulate the expression of the nickel transporter system [64]. The presence of these genes in *M. radiotolerans* MAMP 4754, and their ion metal transport function, suggests their involvement in the heavy metal tolerance, and further supports the isolates’ potential in microbe-assisted phytoremediation and bioremediation, as previously suggested [65]. This is especially supported by the fact that *Enterobacter cloacae subsp*. *cloacae* ENHKU01, *Enterobacter* sp 638, *Klebsiella pneumoniae* 342, *Pseudomonas putida* W619, and *Serratia proteamaculans* 568 (isolated from hyperaccumulator plants *Capsicum annuum*, maize plants, and *Populus trichocarpa* × *deltoides*) were also found to possess heavy metal detoxification properties [66,67]. Furthermore, plant growth promotion enzymes such as glutathione S-transferase and glutathione-dependent dehydrogenase were also identified in *M. radiotolerans* MAMP 4754 and the other endophytes selected for the genome comparison. These enzymes are reported to play a role in the alleviation of oxidative stress caused by abiotic factors such as salinity and heavy metal toxicity, for example [68]. The identification of these enzymes, including the proteins for metal detoxification, indicates that *M. radiotolerans* MAMP 4754 is directly involved in processes of growth promotion in the host plant.

Various heavy metal concentrations for copper, nickel, and zinc were prepared and the maximum tolerance concentration for each metal was determined and is presented in Table 3. *M. radiotolerans* MAMP 4754 was able to tolerate heavy metal concentrations as high as 4.0, 12.0, and 15.0 mM for copper, nickel, and zinc, respectively. The level of copper, nickel, and zinc tolerance of *M. radiotolerans* MAMP 4754 is significant in comparison with what is reported for other *Methylobacterium* strains [10,69]. This suggests, therefore, the potential to utilize *M. radiotolerans* MAMP 4754 in heavy metal bioremediation processes and indicates an adaptation to life inside the hyperaccumulator plant host, *C. erythrophyllum.* Furthermore, the presence of copper-, nickel-, and zinc-detoxifying proteins is in support of the notion that endophytes and their plant hosts transfer beneficial attributes to each other through the process of horizontal gene transfer [70,71,72].

**Figure 1:**
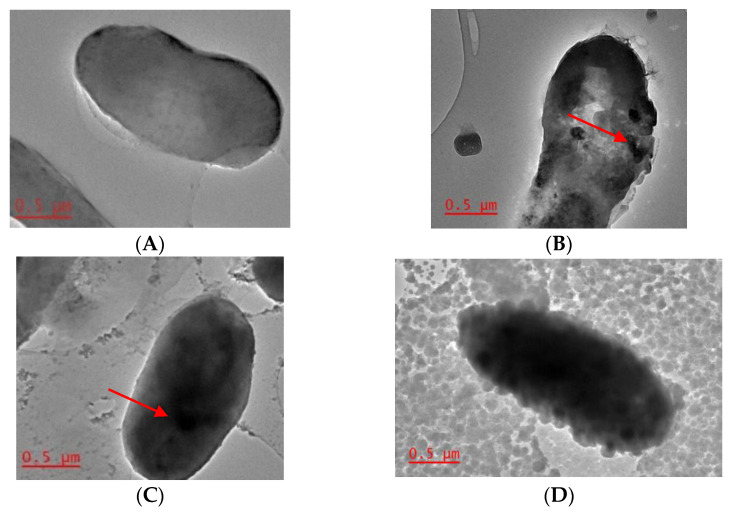
Transmission electron micrographs of *M. radiotolerans* MAMP 4754 after 2 days of inoculation. (**A**): Untreated cell; (**B**): Nickel-treated cells (12 mM); (**C**): Copper-treated cells (4 mM); (**D**): Zinc-treated cells (15 mM). The arrows indicate metal accumulation/aggregation within the cell.

In order to investigate the fate of the treated metals, *M. radiotolerans* MAMP 4754 cells (heavy metal treated and untreated) were viewed under TEM. The transmission electron micrographs show metal accumulation in both the cell wall and the cytoplasmic membrane (Figure 1B–D). Furthermore, cells exposed to high concentrations of zinc showed thickening of the exterior membrane, which alludes to the mechanism of extracellular biosorption of the heavy metal on the cell surface. The localization of the heavy metal aggregates inside the cell suggests that the mediation of metal toxicity is due to the presence of metal-binding, as well as efflux, mechanisms.

As previously discussed by [73], the high resistance of *M. radiotolerans* MAMP 4754 to heavy metal stress is possibly due to an efficient ion efflux and metal complexation and reduction, and thus self-repair capabilities. It may therefore be inferred that metal allocation within bacterial cells is determined by the mechanism of tolerance for each micro-organism [74]. For example, when intracellular sequestration is the method of heavy metal detoxification, the complexation of metal ions occurs in the bacterial cell cytoplasm [75]; extracellular sequestration on the other hand, results in the accumulation of metal ions within the periplasm or the complexation of these heavy metals into less toxic insoluble compounds [75].

### 3.2. M. radiotolerans MAMP 4754 Plant Growth-Promoting Volatiles

For the determination of compounds implicated in plant growth promotion, gas chromatography high-resolution time-of-flight mass spectrometry (GC-HRTOFMS) was performed as described in [40]. Several soil micro-organisms have been directed to promote plant root development based on the capacity of bacterial 1-aminocyclopropane-1-carboxylic acid (ACC) deaminase to hydrolyze and lower the quantity of ACC, an ethylene precursor, in plants [76,77]. They also stimulate plant growth promotion by releasing volatile organic compounds, including esters, aliphatic aldehydes, alcohols, ketones, organic acids, hydrocarbons, and ethers [78,79]. In this study, several volatile organic compounds known to have an influence in plant growth promotion were identified to be produced by *M. radiotolerans* MAMP 4754. These identified bioactive volatile organic compounds have different functional groups, such as acids, ketones, alcohols, sulphides, alkenes, thiazoles, phthalic esters, and phenols (Table 4).

The widely known phenolic defense hormone, salicylic acid, was one of the acids identified in the GC-HRTOFMS analysis. This hormone is known to play a role in several physiological processes in plants, including local and systemic resistance [92]. Furthermore, salicylic acid improves photosynthetic disruption, growth inhibition, abnormal absorption of nutrients, and oxidative disruption that is triggered by heavy metal stress [93,94]. Dimethyl disulphide, which is known to be produced by several plant growth-promoting strains, was also identified. This compound was previously stated to act as a source of sulfur, which contributed to nutrition in tobacco seedlings [89], while [83] indicated its ability to raise biomass in *Arabidopsis thaliana* (2013).

Benzothiazole of the thiazole family was also identified in the GC-HRTOFMS analysis of *M. radiotolerans* MAMP 4754. The compound was previously obtained from various *Pseudomonas* spp., and showed potential for the inhibition of the formation of scleroids caused by the pathogenic fungus *Sclerotinia sclerotiorum,* and increased salt-tolerance levels in soybeans [88,89]. The indole compound, dibutyl phthalate, plus the phenolic compounds (also identified in this study) were previously reported as a requirement for microbial associations within the host plant roots [95]. Eicosene was also identified, and it plays a role in antimicrobial and antifungal activity [87,96]. These results correlate with literature as *Methylobacterium* spp. have been stipulated to have the ability to inhibit plant pathogens, which in turn enhances plant growth promotion [97].

## 4. Conclusions

In conclusion, comparative genome analysis is useful in understanding the genetic diversity of similar or related micro-organisms, and different types of plant interactions including endophyte-assisted phytoremediation. The genome comparison analysis of *M. radiotolerans* MAMP 4754 to other plant growth-promoting endophytes identified proteins such as nickel-responsive transcriptional regulator protein NikR, cobalt–zinc–cadmium resistance protein CzcA, and copper resistance A, which support its role in heavy metal detoxification, a process that can be considered plant promoting for the hyperaccumulator host plant. The maximum tolerance concentrations of 4 mM, 15 mM, and 12 mM for copper, zinc, and nickel, respectively, are significantly high in comparison with other *Methylobacterium* strains, and this indicates the potential for the application of this strain in bioremedial processes. The study also showed that *M. radiotolerans* MAMP 4754 produces plant growth-promoting compounds. The presence of these volatiles suggests that the isolate can be exploited for the discovery of novel products for plant growth, biocontrol, and therapeutics.

## Figures and Tables

**Table 1:** Bacteria used in this study, including Genbank accession number and the nature of their interaction with plants.

Bacterium	Accession Number	Biological Nature	References
*Methylobacterium nodulans* ORS 2060	NC_011894.1	Non-endophytic	[45]
*Methylobacterium mesophilicum* SR 1.6/6	NZ_ANPA01000003.1	Endophyte	[46]
*Methylobacterium radiotolerans* MAMP 4754	NKQS00000000	Endophytic	[47]
*Methylobacterium radiotolerans* JCM 2831	CP001001.1	Endophytic	[48]
*Azospirillum lipoferum 4B*	NC_016622	Endophytic	[49]
*Paraburkholderia phytofirmans* PsJN	CP001053	Endophytic	[50]
*Enterobacter cloacae subsp. cloacae* ENHKU01	CP003737	Endophytic	[51]
*Enterobacter* sp 638	NC_009436	Endophytic	[52]
*Klebsiella pneumoniae* 342	NC_011283	Endophytic	[53]
*Pseudomonas putida* W619	CP000949.1	Endophytic	[54]
*Serratia proteamaculans* 568	NC_009832	Endophytic	[55,56]

**Table 2:** Summary of proteins putatively responsible for heavy metal resistance of copper, nickel, zinc, and siderophore production in *Methylobacterium radiotolerans* MAMP 4754.

Protein_ids	Protein Names	AL	EC	ES	KP	BP	MR	PP	SP
WP_043378427.1	Metallophosphoesterase	1	1	1	1	0	0	1	0
WP_059409106.1	Heavy metal translocating P-type ATPase	1	1	1	1	1	0	1	1
WP_043388287.1	Divalent metal cation transporter	0	1	1	1	1	0	1	0
WP_059409724.1	Siderophore-iron reductase FhuF	0	1	1	1	0	1	1	0
WP_012321357.1	Glutathione S-transferase	1	1	1	1	1	1	1	1
WP_024827434.1	Glutathione-dependent formaldehyde dehydrogenase	1	1	1	1	1	1	1	1
WP_050735140.1	Metal/formaldehyde-sensitive transcriptional repressor	0	1	1	1	0	1	1	0
WP_059409487.1	CusA/CzcA family heavy metal efflux RND transporter	0	1	1	1	0	1	1	1
WP_059408797.1	Amidohydrolase/deacetylase family metallohydrolase	0	1	1	1	0	1	1	0
WP_053621161.1	NRAMP family metal ion transporter	0	1	1	1	0	1	1	0
WP_059409485.1	Heavy metal RND transporter	0	1	1	1	0	1	1	0
WP_059408529.1	Metallophore periplasmic binding protein	0	1	1	1	0	1	1	0
WP_012321311.1	Zinc-dependent hydrolase	1	1	1	1	1	1	1	1
WP_029358139.1	Zinc-binding dehydrogenase	1	1	1	1	0	1	1	1
WP_058608166.1	Zinc ribbon domain-containing protein	0	1	1	1	0	1	1	0
WP_012329798.1	Nickel ABC transporter permease subunit NikA	0	1	1	1	0	1	1	1
WP_012329795.1	Nickel-responsive transcriptional regulator NikR	0	1	1	1	0	1	1	0
WP_059408014.1	Nickel/cobalt efflux protein RcnA	0	1	1	1	0	1	1	0
WP_059408010.1	Nickel resistance protein	0	1	1	1	0	0	1	0
WP_059408014.1	Nickel efflux protein RcnA	0	1	1	1	0	1	1	0
WP_059408075.1	Copper chaperone	0	1	1	1	0	1	1	0
WP_059408113.1	Copper resistance protein A	0	1	1	1	0	1	1	0
WP_059407953.1	Crp/Fnr family transcriptional regulator	1	1	1	1	0	0	1	0
WP_059408521.1	TonB-dependent siderophore receptor	1	1	1	1	1	1	1	1
WP_012319993.1	Multidrug efflux RND transporter permease subunit	1	1	1	1	1	1	1	1
WP_059409802.1	Efflux RND transporter periplasmic adaptor subunit	1	1	1	1	1	1	1	1
WP_029360904.1	Arsenical efflux pump membrane protein ArsB	0	1	1	1	1	1	1	1
WP_059408180.1	Hydrophobe/amphiphile efflux-1 family RND transporter	0	1	1	1	1	1	0	1
WP_012318331.1	MATE efflux family protein	0	1	1	1	1	1	0	0
WP_012321398.1	Fluoride efflux transporter CrcB	0	1	1	1	1	1	0	0

The numbers “1” and “0” in columns 3–10 represent the presence or absence of particular genes in the studied genome, respectively. The reciprocal smallest distance (RSD) algorithm was utilized with the blast E-value (1 E-20) and divergence thresholds (0.2). Strains key: AL is *Azospirillum lipoferum* 4B, EC is *Enterobacter cloacae* subsp. *cloacae* ENHKU01, ES is *Enterobacter* sp 638, KP is *Klebsiella pneumoniae* 342, BP is *Paraburkholderia phytofirmans* PsJN, MR is *Methylobacterium radiotolerans* JCM 2831, PP is *Pseudomonas putida* W619, and SP is *Serratia proteamaculans* 568.

**Table 3:** Maximum tolerable concentration shown by *M. radiotolerans* MAMP 4754 against heavy metals.

HM Concentrations (mM)	1	2	3	4	5	6	7	8	9	10	11	12	13	14	15	16	17	18	19
MTC of Nickel	+	+	+	+	+	+	+	+	+	+	+	+	-	-	-	-	-	-	-
MTC of Copper	+	+	+	+	-	-	-	-	-	-	-	-	-	-	-	-	-	-	-
MTC of Zinc	+	+	+	+	+	+	+	+	+	+	+	+	+	+	+	-	-	-	-

A “+” sign in columns 2–20 indicates the presence of bacterial growth and “-” indicates the absence of bacterial growth.

**Table 4:** Gas chromatography high-resolution time-of-flight mass spectrometry (GC-HRTOFMS) analysis of the crude extract of *M. radiotolerans* MAMP 4754, showing microbial compounds reported for their ability to promote plant growth.

R.T (min:sec)	Molecule Name	MF	Functional Group	References
14:43	Salicylic acid	C_7_H_6_O_3_	Acid	[80]
03:33	2-Butanone, 3-ethoxy-3-methyl-	C_6_H_12_O_2_	Ketone	[81]
17:03	2-Acetoxy-5-hydroxyacetophenone	C_10_H_10_O_4_	Ketone	[82,83]
16:43	2-Propanone, 1,1,1-trifluoro	C_3_H_3_F_3_O	Ketone	[84]
04:26	3-Pentanol, 3-(1,1-dimethylethyl)-2,2,4,4-tetramethyl-	C_13_H_28_O	Alcohol	[81]
24:59	Dibutyl phthalate	C_16_H_22_O_4_	Phthalic esters	[85]
04:00	Propanoic acid, ethyl ester	C_5_H_10_O_2_	Ester	[86]
20:42	Phenol, 2,5-bis (1,1-dimethylethyl)	C_14_H_22_O	Phenol	[86]
25:11	3-Eicosene	C_20_H_40_	Alkene	[87]
16:26	Benzothiazole	C_7_H_5_NS	Thiazole	[88,89]
04:30	Dimethyl, disulfide	C_2_H_6_S_2_	Disulfide	[90]
23:02	3,5-di-tert-Butyl-4-hydroxybenzaldehyde	C_15_H_22_O_2_	Aldehyde	[91]

## Data Availability

The bacterial endophyte reported herein (*M. radiotolerans* MAMP 4754) has been deposited in Genbank with accession number MF133459.
